# Identification of Immune-Related Genes for Risk Stratification in Multiple Myeloma Based on Whole Bone Marrow Gene Expression Profiling

**DOI:** 10.3389/fgene.2022.897886

**Published:** 2022-05-26

**Authors:** Qiang-Sheng Wang, Qi-Qin Shi, Ye Meng, Meng-Ping Chen, Jian Hou

**Affiliations:** ^1^ Department of Hematology, Ningbo Hangzhou Bay Hospital, Ningbo, China; ^2^ Department of Ophthalmology, Ningbo Hangzhou Bay Hospital, Ningbo, China; ^3^ Bone Marrow Transplantation Center, The First Affiliated Hospital, Zhejiang University School of Medicine, Hangzhou, China; ^4^ Department of Hematology, Renji Hospital, Shanghai Jiaotong University School of Medicine, Shanghai, China

**Keywords:** multiple myeloma, immune-related genes, whole bone marrow sequencing, prognostic model, IRF7, SHC1

## Abstract

**Background:** Multiple myeloma (MM) is characterized by abnormal proliferation of bone marrow clonal plasma cells. Tumor immunotherapy, a new therapy that has emerged in recent years, offers hope to patients, and studying the expression characteristics of immune-related genes (IRGs) based on whole bone marrow gene expression profiling (GEP) in MM patients can help guide personalized immunotherapy.

**Methods:** In this study, we explored the potential prognostic value of IRGs in MM by combining GEP and clinical data from the GEO database. We identified hub IRGs and transcription factors (TFs) associated with disease progression by Weighted Gene Co-expression Network Analysis (WGCNA), and modeled immune-related prognostic signature by univariate and multivariate Cox and least absolute shrinkage and selection operator (LASSO) regression analysis. Subsequently, the prognostic ability of signature was verified by multiple statistical methods. Moreover, ssGSEA and GSEA algorithm reveled different immunological characteristics and biological function variation in different risk groups. We mapped the hub IRGs by protein-protein interaction network (PPI) and extracted the top 10 ranked genes. Finally, we conducted vitro assays on two alternative IRGs.

**Results:** Our study identified a total of 14 TFs and 88 IRGs associated with International Staging System (ISS). Ten IRGs were identified by Cox -LASSO regression analysis, and used to develop optimal prognostic signature for overall survival (OS) in MM patients. The 10-IRGs were BDNF, CETP, CD70, LMBR, LTBP1, NENF, NR1D1, NR1H2, PTK2B and SEMA4. In different groups, risk signatures showed excellent survival prediction ability, and MM patients also could be stratified at survival risk. In addition, IRF7 and SHC1 were hub IRGs in PPI network, and the vitro assays proved that they could promote tumor progression. Notably, ssGSEA and GSEA results confirmed that different risk groups could accurately indicate the status of tumor microenvironment (TME) and activation of biological pathways.

**Conclusion:** Our study suggested that immune-related signature could be used as prognostic markers in MM patients.

## Introduction

Multiple myeloma (MM) is a B-cell malignancy characterized by abnormal proliferation of clonal plasma cells in the bone marrow. In recent years, its incidence has been on the rise and it has become the second most common hematologic malignancy (Mireles-Cano et al., 2020; Moser-Katz et al., 2021). MM Patients face multiple inevitable relapses after remission with multidrug combination therapy. The higher the number of relapses, the shorter the remission period and eventually the refractory relapse period, which seriously affects the prognosis (Gerecke et al., 2016). The occurrence of such condition is one of the greatest challenges in the treatment of MM, as it leads to incurable MM. Therefore, it is clinically important to explore the pathogenesis of MM in depth and to discover new therapeutic targets to provide more effective means for the treatment of MM.

Immunotherapy is a new therapeutic option and its efficacy in the treatment of MM needs to be further investigated and improved. The TME is closely related to the immunotherapeutic response (Hou et al., 2019). Studies have shown that dendritic cells (DCs) isolated from MM patients not only have impaired function but also express/produce low levels of key molecules that initiate the immune response, including IL-12, human leukocyte antigen DR (HLA-DR), CD40, CD86, and CD80 (Kawano et al., 2015). The immune checkpoint cytotoxic T lymphocyte-associated protein-4 (CTLA-4) on chromosome two interacts with CD80/CD86 on DCs and negatively regulates the CD28 signaling pathway. Although the killing of MM cells by CD4^+^ T cells is mediated by resident myeloid macrophages (Haabeth et al., 2020), myeloid macrophages in MM is mainly derived from TNF-α and immunosuppressive cytokines IL-10 and IL-1β in the tumor microenvironment, which not only produce angiogenic factors that contribute to tumor growth and invasion, such as vascular endothelial growth factor (VEGF), IL-8, fibroblast growth factor-2, metalloproteinase and cyclooxygenase-2, and colony-stimulating factor-1, but also increase drug resistance in myeloma through direct cell-to-cell interactions (Kawano et al., 2017). PD-L1 is expressed in most MM plasma cells. Increased IFN-γ and toll-like receptor (TLR) ligands induce PD-L1 expression in isolated MM plasma cells (Tamura et al., 2020). Myeloid differentiation factor 88 (MyD88) and TNF receptor-associated factor 6 (TRAAF6) bridging proteins inhibit TLR pathway and suppress not only TLR ligand-induced PD-L1 expression but also IFN-γ-mediated PD-L1 expression (Liu et al., 2007). The above findings suggest that the immune microenvironment plays a key role in MM progression. In this study, we will reveal the abnormal expression of immune-related genes (IRGs) in tumor progression to provide effective diagnosis and treatment for the disease. Nowadays, there have been several studies on the prognosis prediction of MM, such as gene expression inflammatory signature (Botta et al., 2016), EMC-92-gene signature (Kuiper et al., 2012), and genome-wide association studies (GWAS) of MM (Went et al., 2019), etc. Although these study all predict survival status in MM patients, we found that most of research either used CD138^+^ selected cells microarray or mixed samples from various time points. More importantly, prognostic signature based solely on IRGs have not yet been developed in MM patients. Therefore, an in-depth study of the treatment and prognosis of IRGs and individualized immunotherapy is essential to improve the prognosis of MM patients.

In this study, we investigated the potential prognostic value of IRGs in MM by integrating clinical data and pre-treatment gene expression profiling (GEP). Firstly, we identified 102 IRGs and transcription factors (TFs) driving MM progression, and performed gene ontology (GO) and Kyoto Encyclopedia of Genes and Genomes (KEGG) enrichment analyses. Subsequently, immune-related prognostic signature was developed in training cohort and validate in training and testing cohorts. In addition, the protein-protein interaction (PPI) network were extracted 10 Top IRGs. The results of the bioinformatic analysis were supported by the identification of IRF7 and SHC1 genes as hub IRGs, and the vitro assays demonstrated that IRF7 and SHC1 have a function in promoting tumor progression. These results suggested that prognostic signature and hub IRGs may be promising and molecular markers, which in turn provide targets for the diagnosis and prognosis of MM.

## Materials and Methods

### Data Collection and Pre-processing

Whole bone marrow GEP and corresponding clinical features were obtained from The Gene Expression Omnibus (GEO) (https://www.ncbi.nlm.nih.gov/geo/). Importantly, the whole bone marrow samples in the GSE136400 dataset contains five time points, such as before treatment, post Induction, post transplant, post consolidation, and post maintenance. The aim of the study was pre-treatment gene signature prediction, hence we retained only before treatment 354 patients for bioinformatics analysis. Samples were omitted genes with mean expression values less than 0.1 to ensure the significance of the analysis. Detailed clinical information for each sample is provided in Supplementary File S1. We annotated 1,594 TFs and IRGs based on the cis-chromosome and IMMPORT database (http://cistrome.org/CistromeCancer/CancerTarget/; https://www.immport.org/home).

### Weighted Gene Co-Expression Network Analysis (WGCNA)

The ‘WGCNA’ package (Langfelder and Horvath, 2008) screened the genes that were significantly associated with clinical features. According to our previous study (Shen et al., 2021), a soft threshold was determined, an adjacency matrix was clustered, and a hub module was determined. The strongest positive correlation was selected for further analysis by calculating the Pearson correlation coefficient between the modules and International Staging System (ISS). In this study, we classified the transcriptome data into genes modules based on the topological overlap matrix (TOM) and optimal soft threshold (*β* = 7).

### Functional Enrichment Analysis

We used the ‘cluster Profiler’ package for gene ontology (GO) and Kyoto Encyclopedia of Genes and Genomes (KEGG) enrichment analyses of TFs and IRGs involved in disease progression. All MM patients were divided into high- and low-expression groups based on median expression for subsequent GSEA analysis. In addition, we used the c2. cp.kegg.v7.4. symbols and c5. go.v7.4. symbols gene sets from the Molecular Signature Database (MSigDB) for GSEA analysis. The number of permutations was set to 1,000. The criteria for screening statistically significant pathways were set as *p*-value less than 0.05 and FDR less than 0.05.

### Construction and Validation of IRGs-Related Signature

We randomly divided the 354 pre-treatment patients in the GSE136400 dataset by a ratio of 6:4 (caret package in R software). Of these, 214 patients were used as the training set and the remaining 120 patients included in the testing set. In addition, we also added a validation cohort (n = 134), including post maintenance patients GEP. Univariate and multivariate Cox regression analyses were used to investigate the relationship between the expression of IRGs and clinical prognosis in training set. Specifically, we selected genes that were significantly associated with clinical prognosis (*p* < 0.05). Subsequently, the LASSO-Cox regression method was used to select the IRGs involved in the prediction model from the above IRGs. In the training and testing cohorts, the risk score of each individual was analyzed by regression coefficients and their expression in multivariate Cox analysis. MM patients in different sets were classified according to median risk score in training set and survival analysis was used to compare the clinical prognosis of high-risk and low-risk patients. The diversity of clinical information between the different risk groups and the prognostic significance were assessed. The accuracy of the prognostic model was verified using ROC curves with *p* < 0.05 as the significance criterion.

### Comprehensive Analysis of Signature

Cox regression analysis was used to assess the independent prognostic value. We analyzed differences in risk subgroups and clinical characteristics. In addition, we used the ‘rms’ package to construct nomogram containing each IRG. The assessment of the accuracy of model was achieved. In addition, we performed a two-dimensional principal component analysis (PCA) to explore the differences in the discrete state distributions of different risk groups. We combined the top10 genes in the PPI network with prognosis-related IRGs from univariate Cox regression analysis in the entire cohort to obtain two hub IRGs by Venn plot.

### Immune Infiltration Assessment

The ‘GSVA’ package in R software was used to perform a gene set enrichment analysis ssGSEA algorithm to unambiguously present the infiltrating score of 29 tumor-infiltrating immune cells and pathways (aDCs, APC co-inhibition, APC co-stimulation, B cells, CCR, CD8^+^ T cells, Check-point, Cytolytic activity, DCs, HLA, iDCs, Inflammation-promoting, Macrophages, Mast cells, MHC class I, Neutrophils, NK cells, Parainflammation, pDCs, T cell co-inhibition, T cell co-stimulation, T helper cells, Tfh, Th1 cells, Th2 cells, TIL, Treg, Type I IFN Response, and Type II IFN Response). Also, the relationship between the risk subgroups and parameters related to immune cell infiltration in MM was explored.

### CCK-8 Cell Proliferation Detection

RPMI8226 and MM1S cells (2000 cells/well) were inoculated in 24-well plates and cultured for 24 h using Cell Counting Kit-8 (CCK-8 Kit) from Beyotime (Shanghai, China) (sort code C0037). CCK-8 Cell Proliferation Assay Kit (C0037) was purchased from Beyotime (Shanghai, China) and cells were assayed according to its instructions viability. The human MM cell lines RPMI8226 and MM1S were donated by the Department of Hematology, Renji Hospital, Shanghai Jiaotong University School of Medicine, China.

### EdU Detection

EdU-488 cell proliferation assay kit Beyotime (catalog number C0071S), RPMI8226 and MM1S cells (104 cells/well) were placed in 24-well plates using BeyoClick™ and cultured for 24 h. Cell proliferation capacity was detected using the EdU-488 Cell Proliferation Assay Kit (C0071S) purchased from Beyotime (Shanghai, China) according to the instructions.

### Western Blot

RPMI8226 and MM1S cells (5×10^5^ cells/well) were inoculated in 6-well plates and cultured for 24 h. After transfection and growth to 95%, cells were lysed and harvested, and protein concentrations were determined. Primary antibodies and their dilution working solutions were as follows: anti-IRF7 (1:1000), SCH1 (1:1000), HRP-conjugated secondary antibody (1:2000). The Ultra Enhanced ECL kit (G3308, GBCBIO) was used to amplify the exposure signal for western blot (WB) analysis. Grayscale analysis of WB bands was performed using ImageJ software.

### qPCR

Total microarray was extracted from hepatocellular carcinoma cells and tissues using the Total microarray Extraction Kit (R4107; GBCBIO, Guangzhou, China). then, the microarray concentration was measured by nanodrop. Transcript First Strand cDNA Synthesis Kit (0489703000; Roche) was used for the reverse transcription reaction of microarray. Finally, qRT PCR of IRF7 and SCH1 was performed using the Light Cycle 480 SYBR Green I Master Kit (04707516001; Roche) on a Light Cycle 480®II instrument with internal microarray control for GAPDH. we used the 2-ΔCT method to infer the relative expression levels of microarray. All primers for microarray are listed below:

IRF7: F primer-CTTCGTGATGCTGCGGGATA, R primer-TTCTCGCCAGCACAGCTC, Product length 85bp. SHC1: F primer-AGGTCCAACCAGGCTAAGGG, R-primer: GGG​GGC​AGG​AGA​TCC​ATA​GT, Product length 120bp.

### Statistical Analysis

All statistical analyses were performed using the R software (v.4.0.1). The Wilcoxon test was applied for pairwise comparisons. The Kaplan-Meier analysis with the log-rank test was adopted for overall survival comparisons. More detailed statistical methods for transcriptome data processing are covered in the above section. *p* < 0.05 was considered statistically significant.

## Results

### Data Pre-processing

**FIGURE 1:**
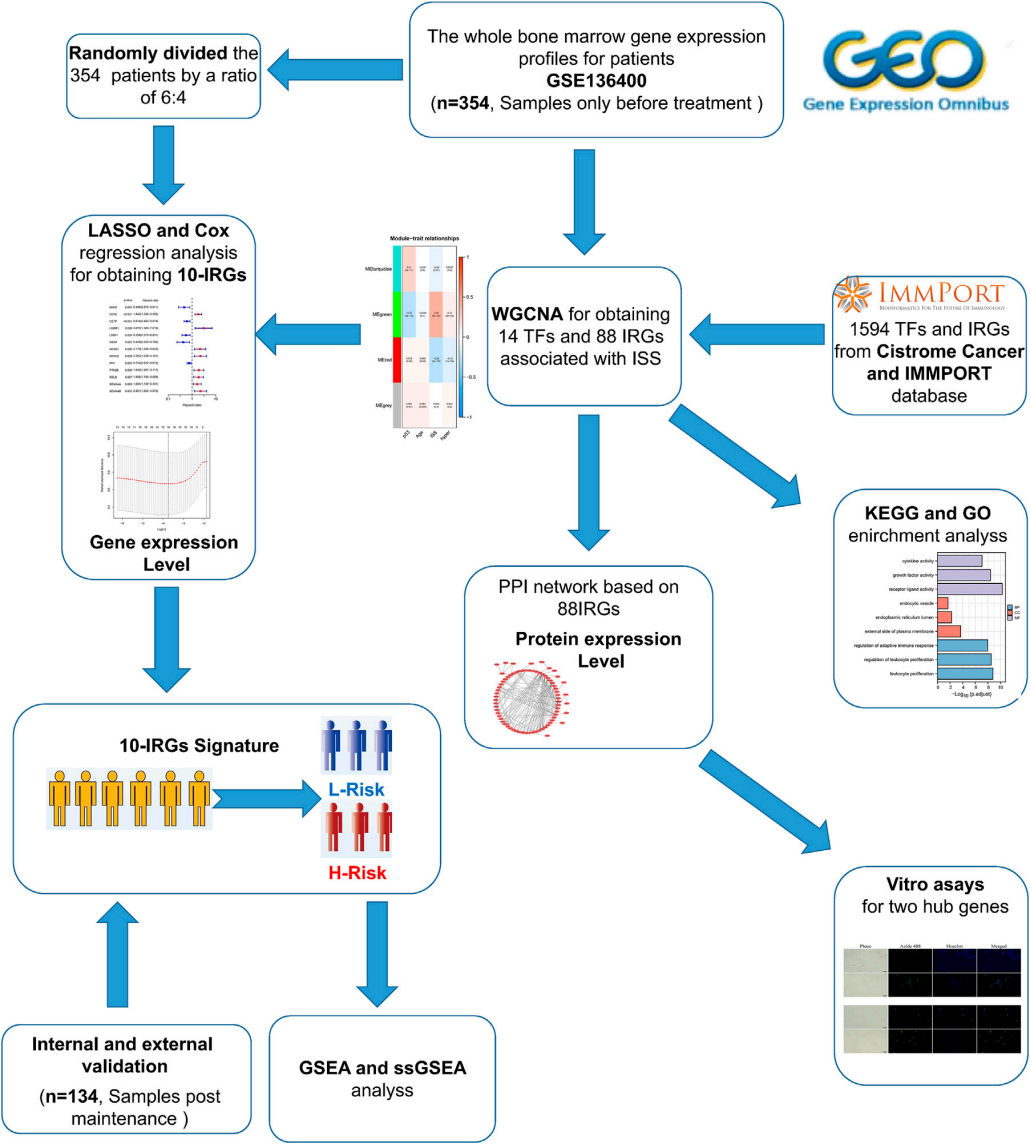
Flaw chart.

The flow chart of our study was shown in Figure 1. To investigate the immune-related features of MM and their prognostic associations, we downloaded the whole bone marrow transcriptome microarray dataset and clinical information of MM patients from the GEO database. Subsequently, we randomly divided the 354 pre-treatment patients in the GSE136400 dataset by a ratio of 6:4. Of these, 214 patients were used as the training set and the remaining 120 patients included in the internal validation set. Bioinformatics analysis was subsequently performed.

### Identification of MM Progression-Related IRGs and TFs

**FIGURE 2:**
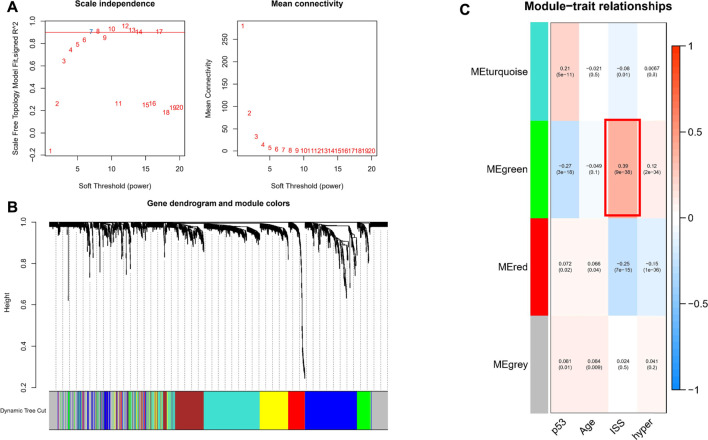
WGCNA of all samples. **(A)** Soft threshold was identified by scale independence and mean connectivity. **(B)** Transcriptome data was classified into different modules. **(C)** Association between the modules and clinical traits.

**FIGURE 3:**
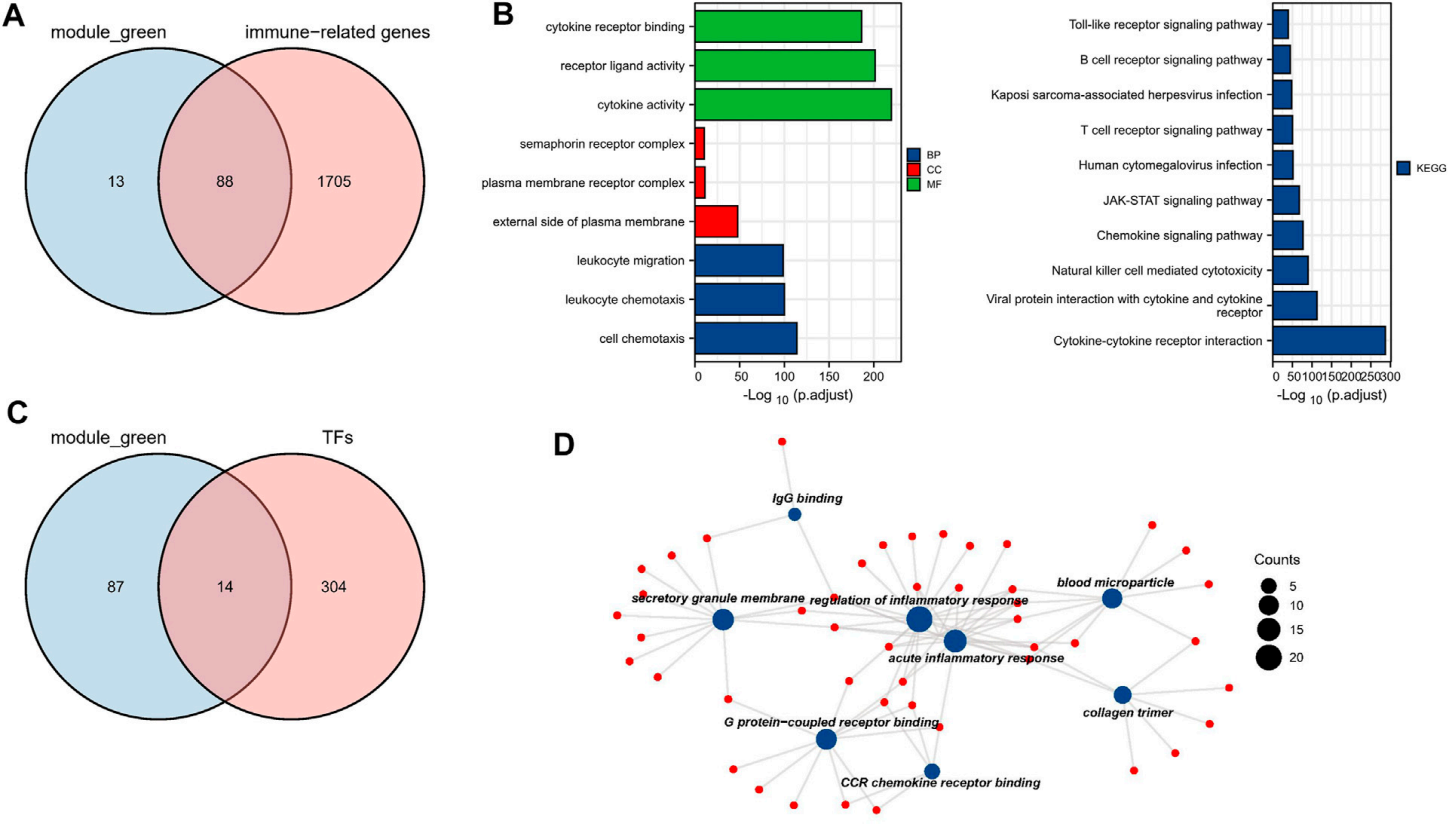
Biological function analysis of TFs and IRGs. **(A)** The venn plot of green module and IRGs. **(B)** IRGs for GO and KEGG pathway enrichment analysis **(C)** The venn plot of green module and TFs. **(D)** The main biological process of TFs enrichment.

To investigate genes that may be involved in MM disease progression, we focused on IRGs and TFs, as they may play a major role in tumor progression. We constructed WGCNA for all patient samples and excluded one sample (GSM4045581) based on the clustering. We classified the transcriptome data into genes modules based on the topological overlap matrix (TOM) and optimal soft threshold (*β* = 7) (Figures 2A,B). The TOM was obtained from: the adjacency matrix (matrix of weighted correlation values between genes) was converted to a topological overlap matrix to reduce noise and false correlation, and the new distance matrix was obtained. Subsequently, we calculated the correlation between modules and clinical features using Pearson method. The genes of the entire data were divided into four modules, with the green module being the hub module for International Staging System (ISS) (r = 0.39, *p* < 0.05) (Figure 2C). Moreover, the classification categories from WGCNA present different proportions of cell types. Most of all, green module had the strongest positive correlation with Treg (r = 0.57), and the strongest negative correlation with macrophages (r = -0.65), as shown in Supplementary Figure S1. We overlapped the genes in the green module with the known TFs and IRGs of the database, 101 TFs and IRGs associated with ISS were identified, which included 88 IRGs (Figure 3A) and 14 TFs (Figure 3C). This result suggested that these 88 IRGs and 14 TFs may drive disease progression in MM.

### Functional Enrichment Analysis

To investigate the biological functions of these 101 TFs and IRGs that may be involved in disease progression, GO and KEGG analysis were performed on the above-mentioned IRGs and TFs. GO analysis showed that genes were enriched mainly in cytokine activity, receptor ligand activity, and leukocyte migration, etc. (Figure 3B). In KEGG analysis, genes were mainly enriched in immune-related pathways such as cytokine-cytokine receptor interaction and natural killer cell mediated cytotoxicity (Figure 3C), suggesting that these genes may influence tumor immunity and thus regulate MM progression. Meanwhile, TFs were mainly enriched in biological processes such as IgG binding, G protein-coupled receptor binding and CCR chemokine receptor binding (Figure 3D), and their functions were highly diversified, suggesting that these TFs may have pro-cancer potential.

### Construction and Validation of Immune-Related Prognostic Signature

**FIGURE 4:**
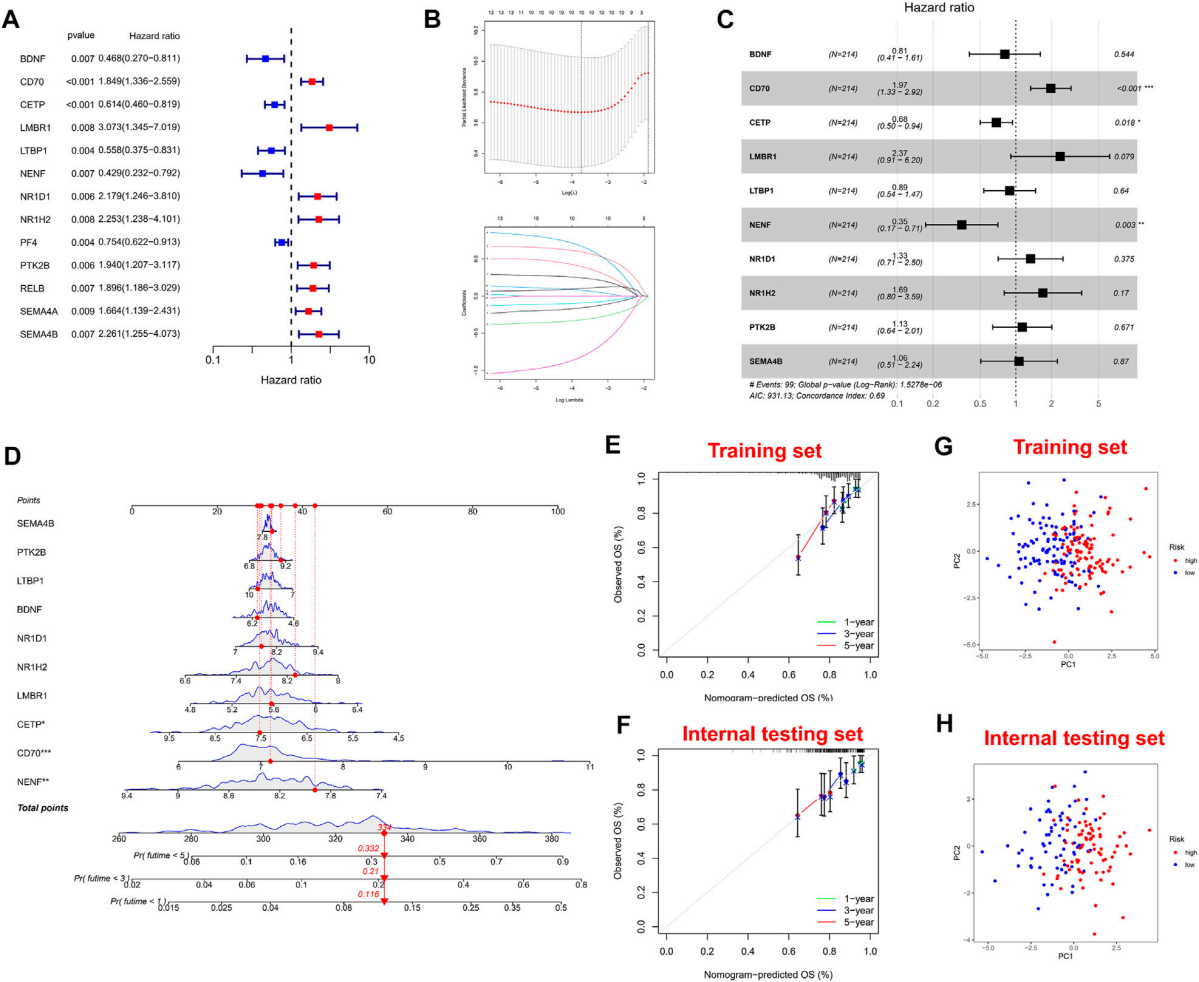
Construction and validation of immune-related signature. **(A)** A forest of univariate cox regression analysis in training set. **(B)** LASSO regression analysis for most suitable λ. **(C)** A forest of multivariate cox regression analysis. **(D)** Nomogram based on 10-IRGs. **(E)** Calibration curve in the training set. **(F)** Calibration curve in the internal testing set. **(G)** PCA plot in the training set. **(H)** PCA plot in the internal testing set. The red dots represent high-risk patients and blue dots represent low-risk patients.

**FIGURE 5:**
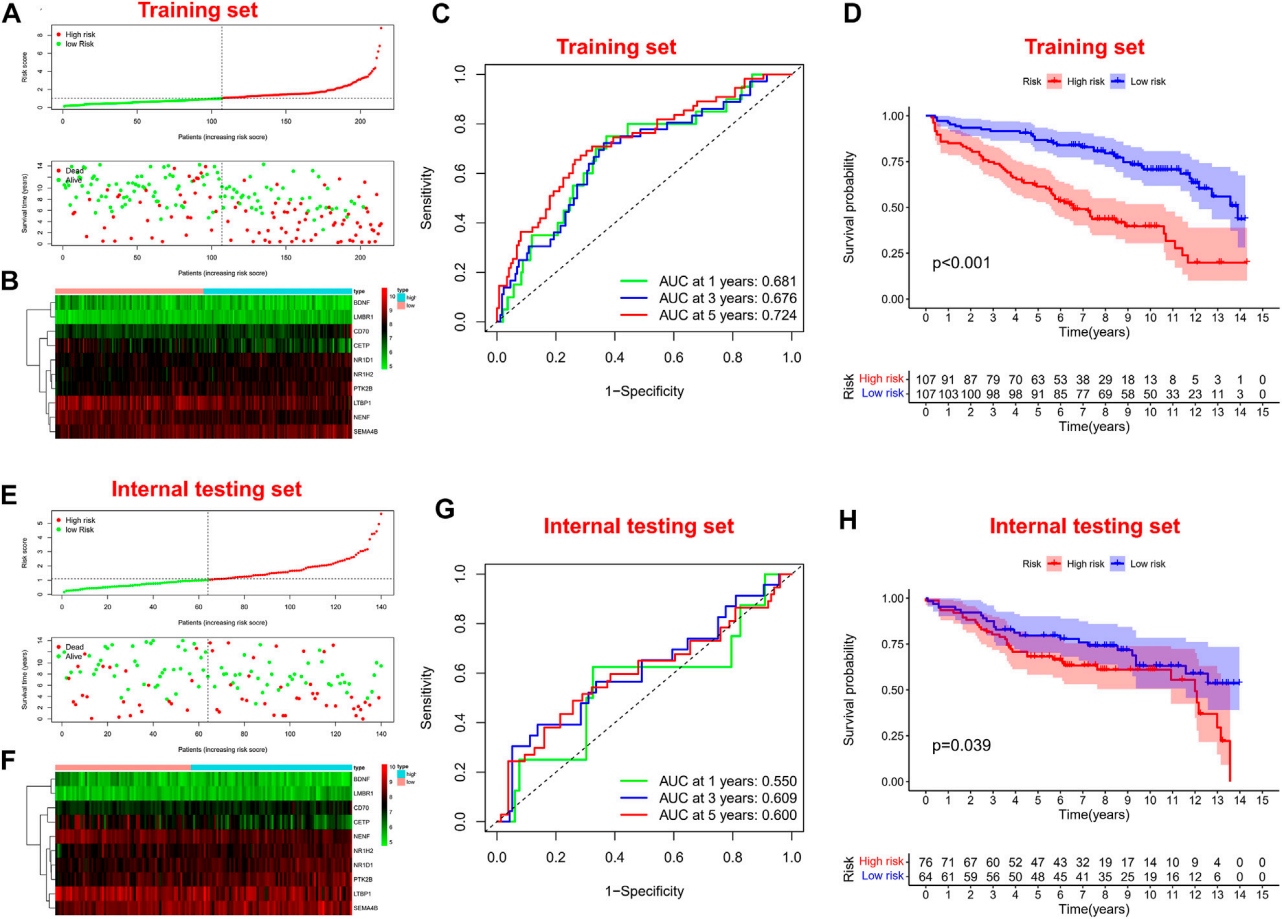
Survival prognostic prediction to test the prognostic model. **(A, B)** Distribution of risk scores, survival status and corresponding gene expression levels of patients in the training set. **(C)** ROC analysis about one, three, and five-year survival prediction in the training set. **(D)** Kaplan–Meier analysis in the training set. **(E, F)** Distribution of risk scores, survival status and corresponding gene expression levels of patients in the internal testing set. **(G)** ROC analysis about one, three, and five-year survival prediction in the internal testing set. **(H)** Kaplan–Meier analysis in the internal testing set.

Firstly, univariate Cox regression analysis was performed on all IRGs to identify potential survival-related IRGs (Figure 4A) in the training cohort. LASSO regression analysis (10-flods) was used to select IRGs to avoid potential over-fitting (Figure 4B). The coefficient of 10-IRGs were identified by multivariate Cox regression analysis and used to develop optimal prognostic characteristics for OS in MM patients (Figure 4C). The risk score formula was obtained based on 10-IRGs = (-0.2138*BDNF) + (0.6791*CD70) + (-0.3790*CETP) + (0.8628*LMBR1) + (-0.1201*LTBP1) + (-1.0512*NENF) + (0.2852* NR1D1) + (0.5262*NR1H2) + (0.1247*PTK2B) + (0.0623*SEMA4B). Subsequently, patients were divided into high-risk and low-risk groups by median risk score. In addition, we constructed a nomogram based on the 10 IRGs (Figure 4D). In both the training and internal testing sets, the calibration curves showed that the one-year, three-year and five-year survival predictions were consistent with the actual observations, indicating that the prediction models were likely to be accurate (Figures 4E,F). Subsequently, we performed PCA analysis to explore the discrete distribution between the high-risk and low-risk groups, and the results indicated that risk profile was able to accurately differentiate patients (Figures 4G,H). To further validate the reliability of the prognostic model, we plotted the distribution of risk scores, survival status and corresponding gene expression levels of the selected individuals in the training (Figures 5A,B) and internal testing sets (Figures 5E,F). In the training set, the AUC values for survival prediction at 1, 3 and 5 years were 0.681, 0.676, and 0.724 (Figure 5C). Kaplan-Meier analysis showed a better prognosis for MM patients in the low-risk group compared to the high-risk group (Figure 5D). In the internal testing set, the AUC values for survival prediction at 1, 3 and 5 years were 0.550, 0.609, and 0.600 (Figure 5G). Kaplan-Meier analysis showed that the risk stratification system was still discriminating for OS in MM patients (Figure 5H), although it may be inappropriate for one-year survival prediction. Moreover, we validated the predictive power of our signature for long-term prognosis in post maintenance patients (n = 134). In another testing set, we also plotted the distribution of risk scores, and survival status (Supplementary Figure S2A). As the risk score increased, more patients died. Especially, for long-term survival prediction, ROC curve analysis showed that risk score had high predictive ability (AUC >0.7) (Supplementary Figure S2B). Moreover, Kaplan-Meier analysis also revealed that the risk stratification system was still discriminating (Supplementary Figure S1C).

**FIGURE 6:**
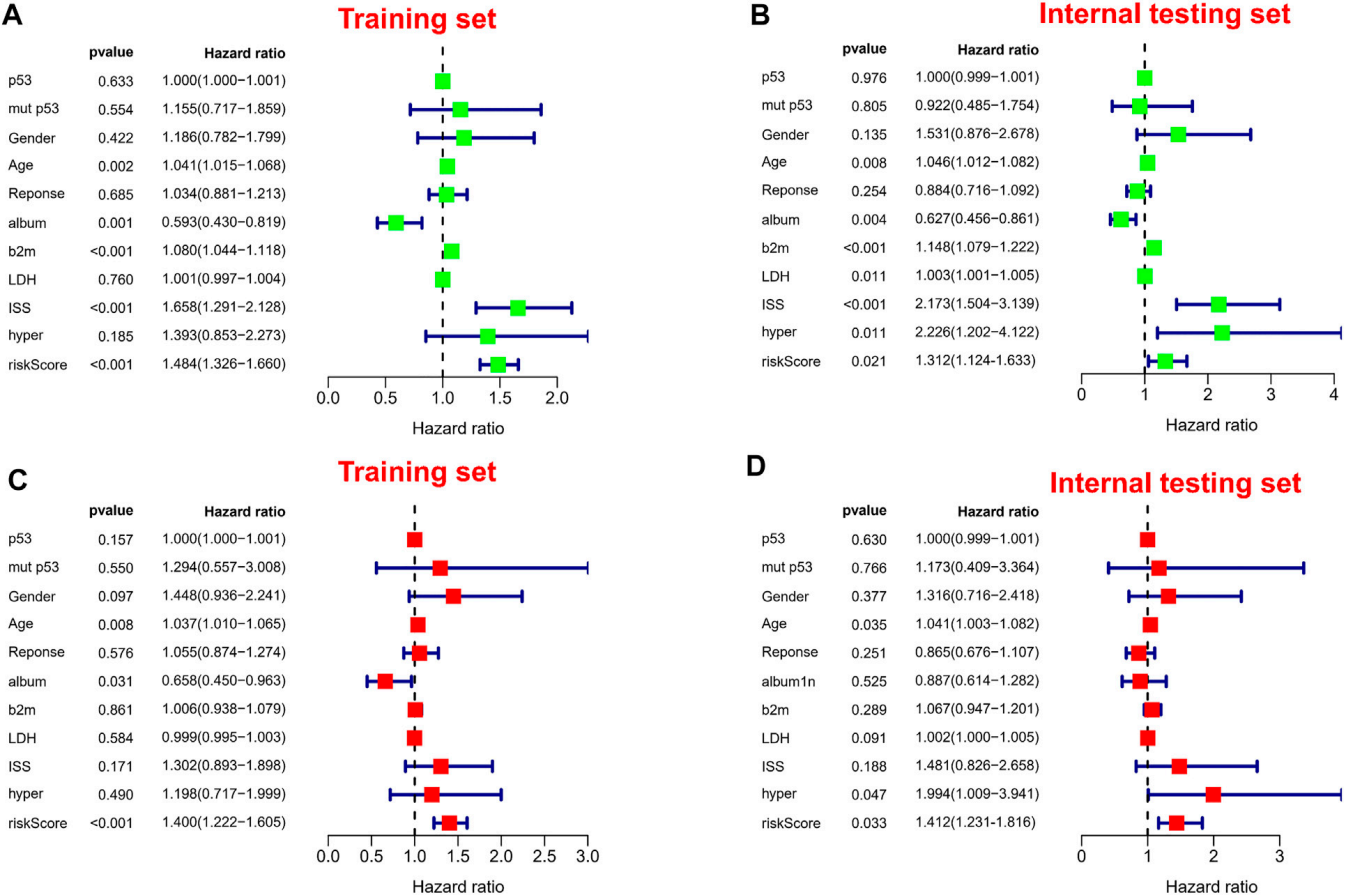
Independent prognostic analysis. **(A,B)** Forest plots of univariate Cox regression analysis in different sets. **(C,D)** Forest plots of multivariate Cox regression analysis in different sets.

It is worth noting we also conducted a web calculator to identify survival possibility in MM patients (http://www.empowerstats.net/pmodel/?m=0_immunesignatureFORmm). To assess the independent prognostic value in the prognostic model, we performed univariate and multivariate Cox regression analyses. Risk score was associated with OS in MM individuals in either the training or validation set (Figures 6A,B). Similarly, risk score was an independent prognostic factor for survival in MM patients (Figures 6C,D). Subsequently, in entire cohort, we explored the correlation between risk scores and clinicopathological parameters. The results showed that our risk scores were significantly correlated with age (Supplementary Figure S3A), ISS (Supplementary Figure S3B), p53 mutation status (Supplementary Figure S3C), albumin (Supplementary Figure S3D), β2-MG (Supplementary Figure S3E), and LDH (Supplementary Figure S3F). The feasibility of progression to advanced tumors gradually increased with increasing risk score, suggesting risk score could be as a indicator in MM progression.

### Immune Infiltration and Biological Pathways in Patients With Different Risk

**FIGURE 7:**
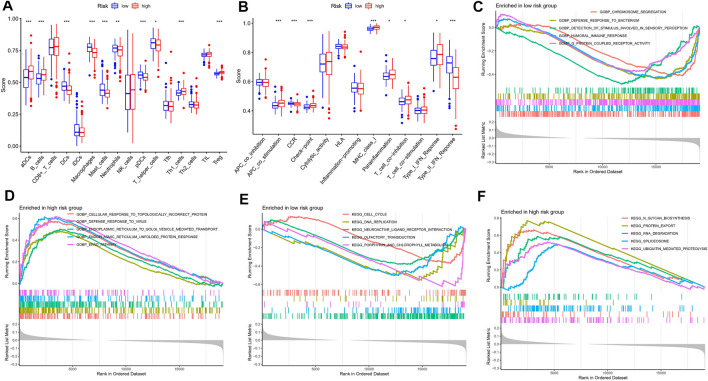
Immune infiltration analysis and GSEA. **(A)** A box plot showed difference of 21 immune cells in different risk subgroups. **(B)** A box plot showed difference of eight immune related pathways in different risk subgroups. **(C,D)** GSEA analysis in the high-risk group. **(E,F)** GSEA analysis in the low-risk group.

A growing number of studies suggest that the tumor microenvironment has an important and essential role in the response to immunotherapy. The tumor microenvironment can be reflected in the type and number of immune cells in the tumor. To further understand the relationship between risk characteristics, we used ssGSEA algorithm to explore the TME in MM. Notably, risk scores were significantly associated with 10 immune cell types, including aDCs, B cells, and Treg, among others (Figure 7A). 8 relevant immune pathways were significantly associated with the expression of risk scores, including APC co-stimulation, CCR signaling, and immune checkpoints (Figure 7B). The ssGSEA results further confirmed that the risk score could indicate the immune status of the TME. Next, GSEA was used to investigate potential biological pathways differences between high-risk and low-risk MM patients. Humoral immune responses and functional pathways, such as cell cycle and DNA synthesis were significantly enriched in the low-risk group (Figures 7C,E). Biological processes such as viral defense responses, endoplasmic reticulum protein transport and signaling pathways such as microarray degradation and protein transport were significantly enriched in the high-risk group (Figures 7D,F).

### Identify Hub IRGs in PPI Network

**FIGURE 8:**
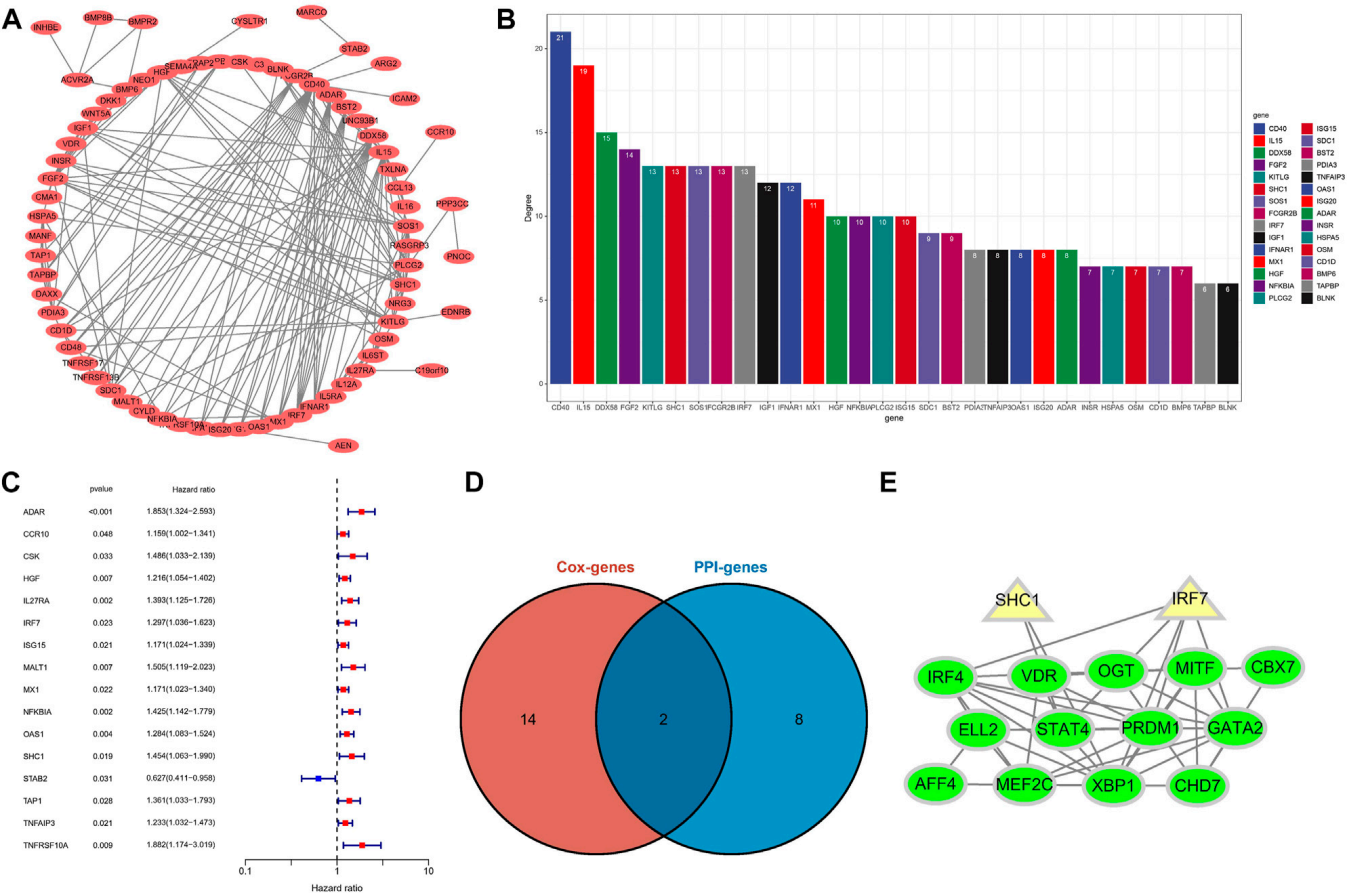
Identification of hub IRGs related to prognosis in PPI network. **(A)** Based on the STRING database, 88 IRGs ring PPI networks are constructed. **(B)** PPI network analyzes topological degree. **(C)** A forest plot of univariate Cox regression analysis in the entire set. **(D)** Venn plot of the Top10 IRGs of PPI network and prognostic-related IRGs. **(E)** Analysis of the interaction between TFs and core IRGs.

To identify potential interaction networks in protein level between 88 IRGs, a circular PPI network (STRING database) was mapped using Cytoscape software (Figure 8A). Also, the top 10 IRGs in topology degree were screened (Figure 8B). Subsequently, by Cox regression analysis based on 88 IRGs, we found a total of 16 IRGs significantly associated with OS for the entire cohort (Figure 8C). Then, we overlapped the Top10 genes in the PPI network and prognostic genes (Figure 8D). Finally, two genes overlapped at the Venn plot, including IRF7 and SHC1. Hence, the above two IRGs were identified as hub IRGs associated with prognosis in protein-protein interaction level. In addition, TFs were identified as important molecules directly regulating the expression of other genes. Therefore, we explored the potential interactions between the 14 TFs in WGCNA and the hub IRGs (IRF7 and SHC1). Excitingly, the results suggested that interactions between TFs and hub IRGs indeed exist (Figure 8E).

### IRF7 and SHC1 Promote Tumor Cell Proliferation

**FIGURE 9:**
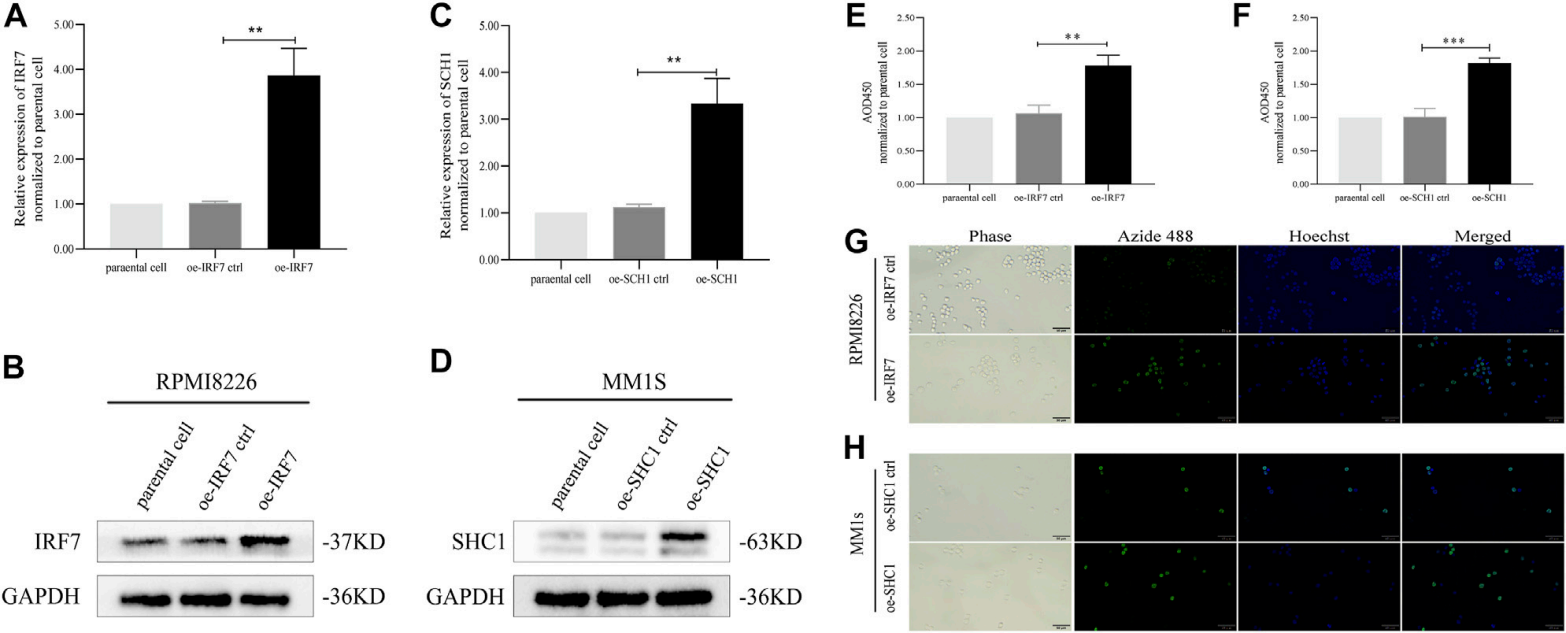
IRF7, SHC1 promote tumor cell proliferation. **(A–D)** qPCR experiment and Western Blot experiment to detect the expression of IRF7 and SHC1. **(E–H)** CCK8 immunofluorescence staining and EDU test to detect the proliferation of tumor cells in the experiment and the control group MM. Protein expression was determined by western blotting and representative results from one of the three independent experiments are presented. Bar graphs were average of experimental replicates from three independent experiments. Error bars represent mean ± s.d.; **p < 0.01; ***p < 0.001; by unpaired two-sided Student’s t-test.

The results of our analysis suggest that IRF7 and SHC1 genes are core IRGs associated with MM prognosis and may be key factors in MM disease progression. To validate this analysis, we overexpressed IRF7 and SHC1 in MM cell lines *in vitro*. QPCR experiments and Western Blot assays verified the successful overexpression of IRF7 and SHC1 (Figures 9A–D). CCK8 immunofluorescence staining and EDU experiments showed that compared to controls, overexpression of both IRF7 and SHC1 promoted tumor cell proliferation (Figures 9E–H). These results support the conclusions of our bioinformatic analysis.

## Discussion

As a clonal plasma cell abnormal proliferative tumor in the bone marrow, MM is accompanied by the secretion of large amounts of M proteins and is highly heterogeneous, leading to symptoms such as hypercalcemia, renal damage, anemia, bone destruction, and pathological clinical signs (Yanai et al., 2012; Corre et al., 2021). With the advent of novel drugs such as immunomodulators and proteasome inhibitors, the prognosis of MM patients has improved significantly, but patients are still repeatedly admitted to hospital for relapse and progression, so multiple myeloma remains an incurable type of disease. Therefore, it is clinically important to explore new molecular biological markers to track the treatment effect of MM, predict the disease progression, and provide more effective treatment options for MM. In our study, we screened out 10-IRGs involved in signature (BDNF, CETP, CD70, LMBR, LTBP1, NENF, NR1D1, NR1H2, PTK2B and SEMA4) and two hub IRGs in PPI network (IRF7 and SHC1). *In vitro* experiments showed that IRF7 and SHC1 could promote the proliferation of MM cell lines. It is suggested that IRF7 and SHC1 may play an important role in promoting the progression of MM. We believed that the above 12 novel markers could provide more possibilities for future MM therapies.

IRF7 is a major regulator of viral immune responses, which is type I interferon-dependent and tumorigenic (Lan et al., 2019). IRF7 not only affects tumor growth and malignant transformation of various tumor populations, but also regulates the development of myeloid-derived suppressor cells in cancer (Robak et al., 2018). Previous reports have shown that IRF7 is highly necessary for monocytes to differentiate them from macrophages (Lu and Pitha, 2001). In IRF7-deficient mice, it has potential effects on the accumulation of immature myeloid cells and on the dynamics of IRF7 expression in myeloid cell differentiation. Factors from tumors can prevent IRF7 expression in myeloid progenitor cells, which may lead to the accumulation of G-MDSC (Yang et al., 2017). Targeting IRF7 may help to reverse the abnormal differentiation of myeloid cells and thus play a role in tumor immunotherapy. This suggests that IRF7 may be the key to MM immunotherapy. Overexpression of SHC1 promotes activation of MM cell lines and progression of MM. the SHC1 gene encodes an adaptor protein that is an important regulator of several tyrosine kinase signaling pathways. In other oncology studies, it has been suggested to promote immunosuppression and is a key regulator of breast cancer (Ahn et al., 2017). Furthermore, overexpression of SHC1 is associated with low survival rates in stage IIA colon cancer (Grossman et al., 2007). Previous studies have suggested that SHC1 associated with imbalance in integrin expression may be a prognostic predictor of clear cell renal cell carcinoma (ccRCC) (Lu et al., 2016). Interestingly, in our present study, SHC1 was an important hub IRGs in the PPI network, suggesting that SHC1 may play a general broad-spectrum function in tumor progression.

In recent years, it has been found that the bone marrow microenvironment plays a key role in the development of MM. The bone marrow microenvironment is composed of immune cells, fibroblasts, bone marrow-derived inflammatory cells and lymphocytes. Under normal conditions, natural killer cells (NK cells) and cytotoxic1 lymphocytes are present in the bone marrow environment and can exert a powerful anti-tumor response. However, the immunosuppressive microenvironment arises due to the presence of tumor cells, which can be of great benefit in expanding the immunosuppressive cell population (Haabeth et al., 2020). A better understanding of the tumor microenvironment can help to determine the prognostic value and therapeutic outcome of MM patients. Immunotherapy is an important and effective treatment for a large number of tumors, and IRGs are closely associated with tumor progression (Murray and Anagnostou, 2021). Currently, MM remains a difficult area of treatment due to recurrence and repeated hospital admissions. Therefore, the discovery of a more powerful tool is an urgent need, and immunotherapy has become a new focus of public attention. Although there has been an increasing number of studies on the relevance of immunotherapy to MM in recent years, more in-depth basic exploration and clinical trials are still needed to apply IRGs to clinical diagnosis and treatment. In our study, we developed a IRGs signature, and the important role of our signature in prognosis was confirmed by various statistical methods. In both the training and testing sets, the calibration curves showed that the one-year, three-year and five-year survival predictions were consistent with the actual observations. PCA analysis to explore the discrete distribution between the high-risk and low-risk groups, In the training set, the AUC values for survival prediction at 1, 3 and 5 years were 0.681, 0.676, and 0.724. Kaplan-Meier analysis showed a better prognosis for MM patients in the low-risk group compared to the high-risk group. Moreover, we validated the predictive power of our signature for long-term prognosis in post maintenance patients (n = 134). As the risk score increased, more patients died. Especially, for long-term survival prediction, ROC curve analysis showed that risk score had high predictive ability. Compared with CD138^+^ selected cells microarray, whole bone marrow microarray is cheaper and easier to promote to clinical practice (Kuiper et al., 2012; Botta et al., 2016; Went et al., 2019). In our study, ssGSEA algorithm was used to show the changes of immune cells and immune function using the whole bone marrow samples. These are all analyses that CD138 + selected cells microarray can’t do.

However, there are numerous limitations to our study that should be considered. Firstly, our research was only based on the GSE16400 dataset, and only pre-treatment whole bone marrow GEP can be used for survival prediction. More independent data sets are needed to verify the risk model we identified. When extending our findings to different treatment or GEP, caution is advised. Moreover, two hub genes in PPI network were validated *in vitro*, and the other 10-IRGs were not further explored. Hence, we will need to conduct more experiments in the future to confirm our conclusion. In conclusion, our study identified a risk model associated with MM prognosis through a series of bioinformatics analyses, and this risk score may have important implications for MM progression.

## Data Availability

The datasets presented in this study can be found in online repositories. The names of the repository/repositories and accession number(s) can be found in the article/Supplementary Material.
