# Supplementary figures and images for: Identification of Immune-Related Genes for Risk Stratification in Multiple Myeloma Based on Whole Bone Marrow Gene Expression Profiling
Source: Front Genet. 2022 May 26;13:897886. doi: 10.3389/fgene.2022.897886 (PMC9178200; doi:10.3389/fgene.2022.897886)

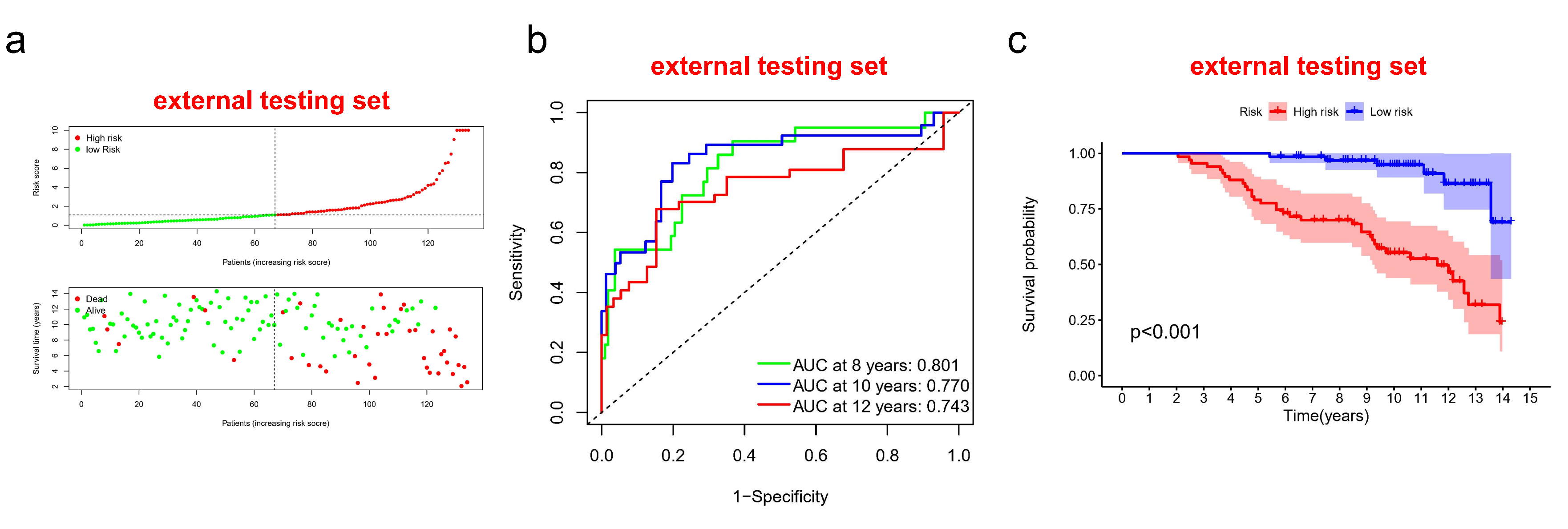

Supplement: Supplementary file 1 [file Image1.TIFF]

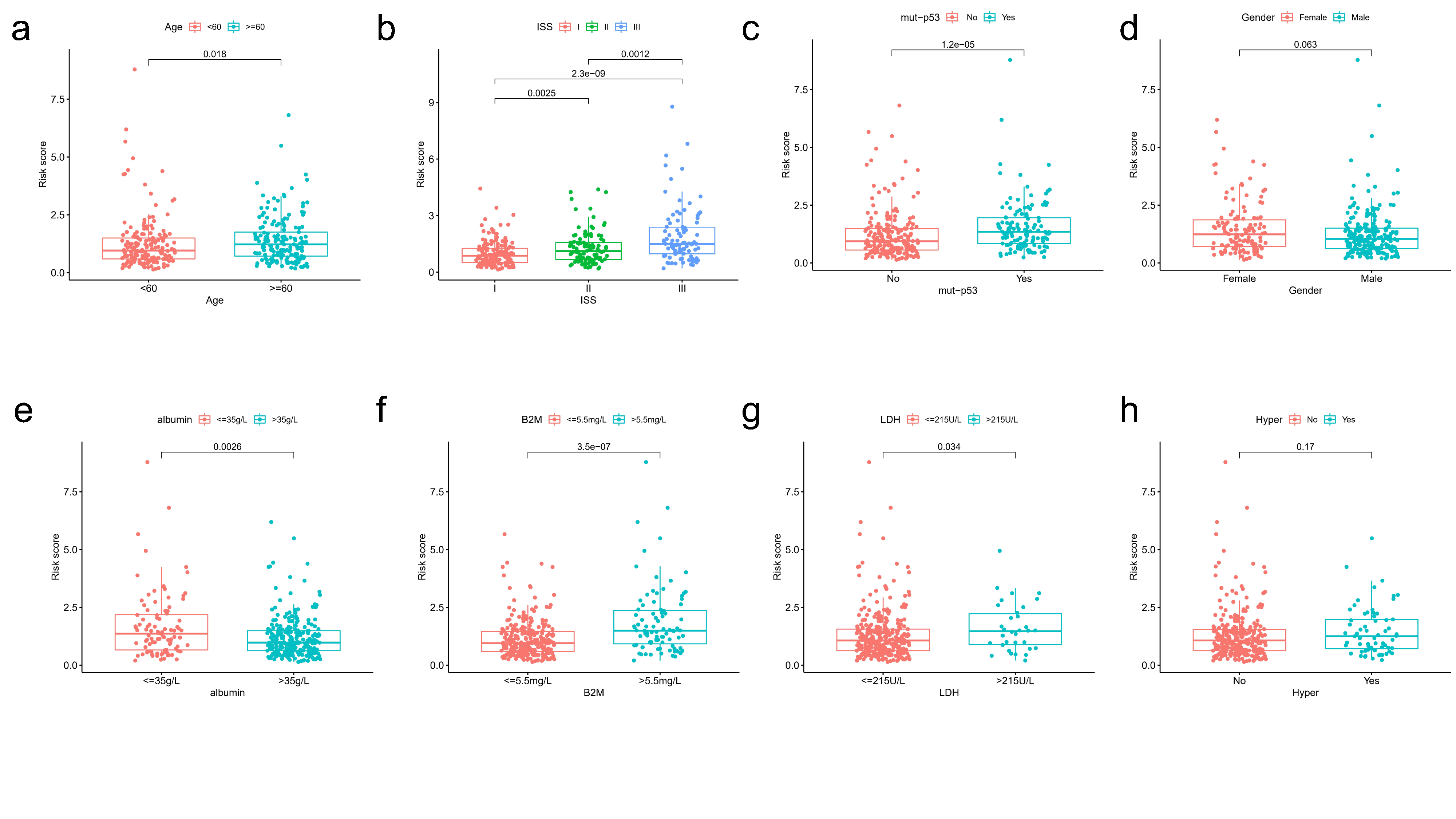

Supplement: Supplementary file 3 [file Image2.TIFF]
